# Intersecting Realms of Skin and Neurology in Systemic Lupus Erythematosus: A Systematic Review of Dermatological and Neurological Manifestations and Their Impact on Long-Term Prognosis

**DOI:** 10.7759/cureus.53142

**Published:** 2024-01-29

**Authors:** Sergio Rodrigo Oliveira Souza Lima, Ayah E Elamin, Hamza Al Balushi, Khalid Shahzad, Shariq K Baluch, Faraz A Khan, Abdullah Shehryar, Abdur Rehman, Biniyam J Batu, Biruk D Ayalew, Noor Abdullah Yahya, Han Grezenko

**Affiliations:** 1 Plastic Surgery, Hospital da Bahia, Salvador, BRA; 2 Anatomy, National University, Khartoum, SDN; 3 Medicine, First Bethune hospital, Muscat, OMN; 4 Internal Medicine, King Edward Medical University, Lahore, PAK; 5 Internal Medicine, Universidad Autónoma de Guadalajara, Guadalajara, MEX; 6 Pediatrics, Hind Institute of Medical Sciences, Sitapur, IND; 7 Dermatology, Allama Iqbal Medical College, Lahore, PAK; 8 Surgery, Mayo Hospital, Lahore, PAK; 9 General Practice, St. Paul's Hospital Millennium Medical College, Addis Ababa, ETH; 10 Internal Medicine, St. Paul's Hospital Millennium Medical College, Addis Ababa, ETH; 11 Family Medicine, Dubai Medical College, Dubai, ARE; 12 Neurology, Barrow Neurological Institute, Phoenix, USA

**Keywords:** long-term prognosis, npsle, neuropsychiatric sle, neurological manifestations, dermatological manifestations, sle, systemic lupus erythematosus

## Abstract

Systemic Lupus Erythematosus (SLE) is a complex autoimmune disease characterized by diverse manifestations, notably in dermatological and neurological domains. This review aims to synthesize the current understanding of these manifestations and their impact on long-term prognosis.

Adhering to PRISMA guidelines, we conducted a comprehensive search across multiple databases, focusing on studies exploring SLE's dermatological and neurological aspects. Selected studies were analyzed to understand their epidemiology, pathophysiology, clinical presentation, and impact on prognosis.

Six pivotal studies were reviewed, highlighting the severity of neuropsychiatric SLE, the progression of skin diseases, and their systemic implications. Notably, studies underscored the role of high disease activity and specific antibodies in the development of neuropsychiatric symptoms and the progression of cutaneous manifestations.

The review emphasizes the need for an interdisciplinary approach to managing SLE, considering the interplay between its dermatological and neurological manifestations. It suggests that tailored treatment strategies, early detection, and comprehensive care are crucial for improving patient outcomes. This synthesis provides a foundation for future research to develop integrated care protocols and advance patient care in SLE.

## Introduction and background

Systemic Lupus Erythematosus (SLE) is an intricate and multifaceted autoimmune disorder known for its diverse and often debilitating manifestations, particularly affecting dermatological and neurological systems. The disease presents unique challenges in diagnosis and management, largely due to its variability in symptom presentation and the complex interplay of its clinical manifestations.

Epidemiologically, SLE exhibits varied incidence and prevalence rates globally, reflecting the influence of genetic, environmental, and other factors in its development. The comprehensive review by Danchenko et al. [1] underscores this variability, drawing attention to the differing disease burden across various populations. Particularly noteworthy is the increased prevalence and severity of SLE among non-white racial groups, indicating a multifactorial etiology that transcends simple genetic explanations.

Among the myriad symptoms of SLE, neuropsychiatric manifestations stand as particularly complex and challenging. Bertsias and Boumpas [2] delve into the intricacies of diagnosing and treating neuropsychiatric SLE (NPSLE), highlighting the role of heightened disease activity and the presence of antiphospholipid antibodies in its pathogenesis. This complexity is further expanded by Kivity et al. [3], who detail the extensive range of neuropsychiatric symptoms observed in SLE patients, from relatively mild conditions to severe, life-threatening events. Their work emphasizes the significant impact of these symptoms on disease severity and patient's quality of life.

The dermatological aspect of SLE, another critical domain, has been thoroughly investigated in several studies, including those by Nyberg [4] and Tebbe [5]. These studies elucidate the progression of skin diseases associated with SLE and their implications for systemic health. They underscore the importance of regular interdisciplinary follow-ups and a deep understanding of clinical and laboratory markers for more effective disease management.

In the broader spectrum of SLE management, research by Fanouriakis et al. [6] and Aringer et al. [7] provides valuable insights into therapeutic strategies and classification criteria. Their work is essential for addressing both dermatological and neurological symptoms in a cohesive and integrated treatment approach.

This systematic review aims to integrate these diverse research threads to comprehensively understand the interplay between dermatological and neurological manifestations in SLE and their collective impact on long-term prognosis. We aim to deepen the understanding of SLE's complex manifestations to facilitate improved patient care and inform future research directions.

Our primary objective is to consolidate and critically evaluate the existing literature on the dermatological and neurological manifestations of SLE. We aim to present a comprehensive overview of their epidemiology, pathophysiology, clinical presentation, and impact on long-term prognosis. This synthesis is intended to enhance the understanding of these intersecting domains and their implications, thereby aiding in developing more effective treatment strategies and advancing patient care within the context of SLE.

## Review

Material and methods

Search Strategy

Our search strategy, aligned with the PRISMA guidelines, was meticulously designed to comprehensively identify studies relevant to the dermatological and neurological manifestations of SLE. To encompass all pertinent literature, we conducted thorough searches across multiple electronic databases, including PubMed, Medline, Embase, and the Cochrane Library. Our search spanned from the databases' inception to November 2023.

The search incorporated keywords and MeSH terms pertinent to our topic, such as 'Systemic Lupus Erythematosus,' 'neuropsychiatric manifestations,' 'dermatological symptoms,' and 'long-term prognosis.' We employed Boolean operators like 'AND' and 'OR' for an effective combination of these terms, for instance, 'Systemic Lupus Erythematosus AND neuropsychiatric manifestations' or 'dermatological symptoms OR long-term prognosis.'

In addition, we scrutinized the reference lists of the identified articles for further relevant studies. Our strategy was expanded by exploring clinical trial registries and conference proceedings to include unpublished or ongoing research. An expert in medical information retrieval reviewed the search strategy to ensure its adherence to the PRISMA guidelines and comprehensiveness.

Eligibility Criteria

The eligibility criteria for this review have been carefully constructed to ensure rigor and relevance. The inclusion criteria encompass peer-reviewed research articles, cohort studies, and clinical trials focusing on patients diagnosed with SLE. We include studies investigating SLE's dermatological and neurological manifestations, their impact on prognosis, and patient outcomes. Studies that explore treatment approaches and their efficacy in these specific manifestations are also considered. To maintain linguistic consistency, only English-language studies are included.

Conversely, studies not directly exploring SLE's dermatological and neurological aspects are excluded. This ensures the review remains focused and pertinent. We excluded studies based solely on animal models, non-English publications, and grey literature such as conference abstracts, posters, and unpublished works. Any survey with insufficient data on the specified manifestations that don't align with the central theme of intersecting skin and neurology in SLE is also excluded. This approach ensures the inclusion of high-quality, relevant studies that contribute significantly to understanding and managing these specific aspects of SLE.

Data Extraction

The data extraction process for this review is structured to ensure reliability and validity. Initially, articles are screened based on their titles and abstracts. Two independent reviewers assess these for relevance, categorizing them as "relevant," "not relevant," or "probably relevant." This preliminary assessment is crucial for focusing on the most pertinent articles.

Subsequently, full-text articles deemed potentially eligible are thoroughly reviewed. Data extraction from these articles is conducted using a standardized Microsoft Excel form, ensuring uniformity in the data collection process. Each reviewer independently applies the predefined inclusion and exclusion criteria. In discrepancies or disagreements between the reviewers, a third reviewer resolves these issues through discussion, ensuring accuracy and consistency in the selection process.

The data extraction form captures critical information, including the authors, publication year, country of origin, participant characteristics, study settings, study design, outcome measures, and significant results. This detailed approach allows for a comprehensive analysis and ensures that all relevant information is considered for the review.

After the data extraction process, each study was evaluated for its methodological quality using the Newcastle-Ottawa Scale (NOS). The NOS is a comprehensive tool designed for the assessment of the quality of non-randomized studies, particularly cohort and case-control studies. It evaluates studies based on three broad domains: Selection, Comparability, and Outcome. In the Selection domain, studies are judged on the representativeness of the exposed cohort, the selection of the non-exposed cohort, and the ascertainment of exposure. Comparability examines the comparability of cohorts on the basis of design and analysis. Finally, in the Outcome domain, the assessment focuses on the assessment of outcome, the length and adequacy of follow-up for outcome events. Each study was awarded a star rating in these categories, with a maximum possible score of nine stars. This star rating system provided a quantifiable measure of study quality, which was used to ensure the inclusion of high-quality, relevant studies in our review.

Data Analysis and Synthesis

The data analysis and synthesis for this review are designed to comprehensively understand SLE's dermatological and neurological manifestations. Firstly, quantitative data, such as prevalence rates, patient demographics, and outcome measures, will be analyzed using statistical methods appropriate to the data types and study designs. This might involve meta-analysis techniques if the data across studies are sufficiently homogeneous.

For qualitative data, such as patient experiences and treatment approaches, thematic synthesis will be employed. This involves coding the data and identifying recurrent themes across the studies. These themes will help elucidate the broader patterns and insights regarding SLE manifestations.

Quantitative and qualitative findings will be integrated to provide a holistic view of the research question. This integrated synthesis will highlight the collected data's correlations, divergences, and unique insights. It will also include a narrative summary, where the findings are discussed in the context of existing literature, providing a comprehensive understanding of the intersecting realms of skin and neurology in SLE.

The synthesis will also assess the quality and strength of the evidence, noting any gaps or inconsistencies in the literature. This approach will ensure a robust and detailed understanding of SLE's dermatological and neurological aspects, contributing valuable insights to the field.

Results

Study Selection Process

**Figure 1:**
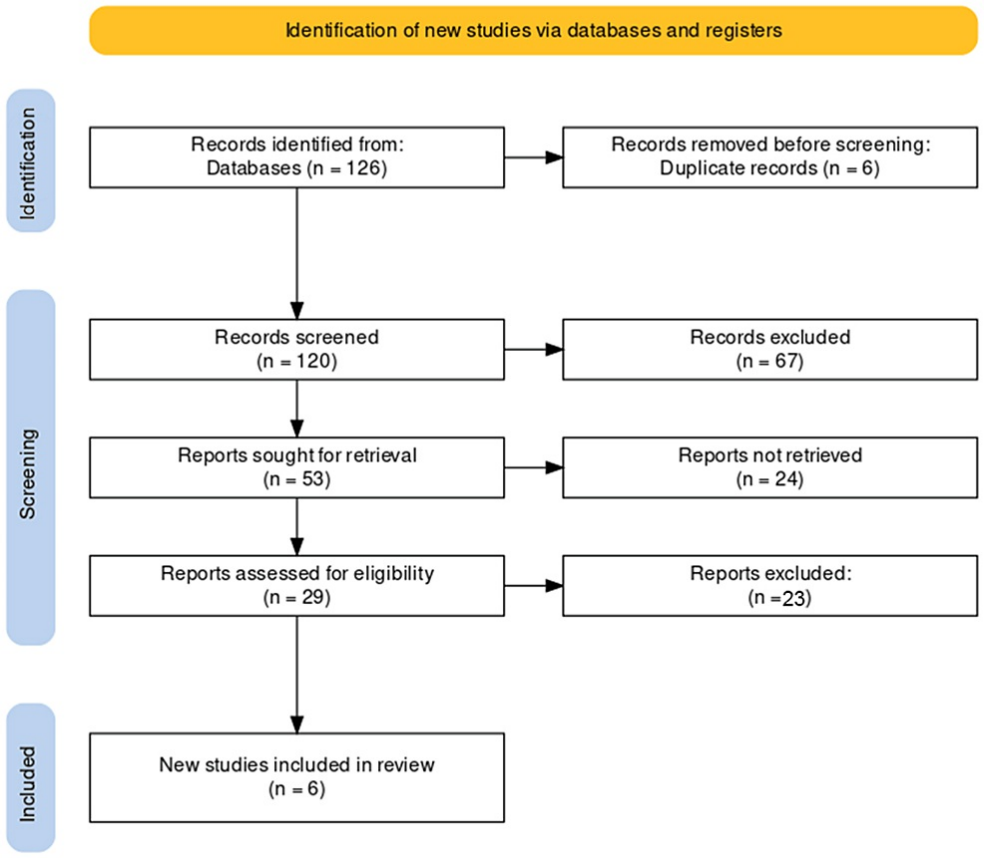
PRISMA flow diagram of the selection of studies for inclusion in the systematic review.

The search across multiple databases yielded a total of 126 articles. After initial screening, six duplicates were removed, leaving 120 articles for title and abstract review. This review led to the identification of 53 potentially relevant studies. These studies underwent a full-text assessment to evaluate their eligibility based on predefined inclusion and exclusion criteria. Ultimately, six studies met the inclusion criteria and were selected for inclusion in the review. The reference lists of the chosen articles did not yield any additional eligible studies. This selection process is illustrated in the PRISMA flowchart, Figure 1, offering a transparent and systematic visual summary of the study selection methodology.

Characteristics of Selected Studies

In our systematic review, we have meticulously analyzed six pivotal studies, each offering distinct insights into the dermatological and neurological aspects of Systemic Lupus Erythematosus. These studies range from retrospective analyses to prospective cohort studies, covering a diverse patient population. Key features, including study design, techniques, duration, sample sizes, and crucial findings, are concisely summarized in Table 1. This table serves as a quick reference to understand the scope and implications of each study, facilitating a comprehensive understanding of SLE's complex manifestations.

**Table 1:** Summary of key findings on neurological and dermatological manifestations in systemic lupus erythematosus (SLE): A comparative analysis of selected studies NPSLE: Neuropsychiatric Systemic Lupus Erythematosus; JSLE: Juvenile Systemic Lupus Erythematosus; SLE: Systemic Lupus Erythematosus; CNS: Central Nervous System; Ro/SSA: Ro/Sjögren's-syndrome-related antigen A; MCTD: Mixed Connective Tissue Disease; LE: Lupus Erythematosus; CDLE: Chronic Discoid Lupus Erythematosus; SCLE: Subacute Cutaneous Lupus Erythematosus

First Author	Year	Study Design	Study Technique	Study Duration	Sample Size	Neurological Findings	Dermatological Findings	Conclusion
Shangzhu Zhang [4]	2021	Retrospective multicenter study	Retrospective data collection, Kaplan-Meier curves, log-rank test, etc.	June 2012 - June 2016	194 patients with NPSLE	Sixteen subtypes of NPSLE were identified; the most common were seizure, acute confusional state, and cerebral vascular disease.	Higher occurrence of typical lupus symptoms like malar rash, oral ulcers, and alopecia in NPSLE patients.	High disease activity and positive anti-ribosomal P protein antibodies may be risk factors for NPSLE.
Sern Chin Lim [5]	2021	Retrospective study	Retrospective analysis of NPSLE patients	January 2004 - May 2017	28 out of 141 JSLE patients	Juvenile-onset NPSLE primarily presented with seizures, delirium, and visual complaints. CNS is involvement common.	Accompanying lupus manifestations included acute cutaneous lupus, hypocomplementemia, nephritis, etc.	Juvenile-onset NPSLE presents diverse clinical features associated with high disease activity. Most patients had a good prognosis.
Nuntana Kasitanon [8]	Not Specified	Retrospective study	Analysis of neuropsychiatric episodes in SLE patients	Not Specified	91 patients with SLE	Predominant neuropsychiatric manifestations were seizures, psychosis, acute confusional state, etc.	More cutaneous vasculitis in NPSLE patients.	Neuropsychiatric manifestations, predominantly seizures and psychosis, are common in SLE patients.
Filippa Nyberg [9]	2008	Prospective cohort study	Questionnaire and clinical examination	Two years	102 Ro/SSA-positive patients	Not specifically reported	Progression in skin disease, including drug-induced subacute cutaneous lupus erythematosus.	New autoimmune diseases and skin disease progression are common in Ro/SSA-positive patients.
Hanan Al Rayes [10]	2002	Retrospective study with prospective follow-up	Follow-up in clinics, laboratory evaluations	1982 - 1999	18 patients with MCTD	Aseptic meningitis, carpal tunnel syndrome, seizure disorder, peripheral neuropathy.	Lupus-like rash, scleroderma-like rash, and cutaneous vasculitis.	The majority of patients had favorable outcomes, with pulmonary hypertension being the most frequent cause of morbidity and mortality.
B Tebbe [11]	Not Specified	Multicenter, retrospective analysis	Documentation and analysis of patients with various forms of LE	1989 - 1994	296 patients with Lupus Erythematosus	Not specifically reported	Chronic discoid LE (CDLE) and subacute cutaneous LE (SCLwere E) were observed.	Specific clinical and laboratory markers can identify the risk of systemic involvement in patients with cutaneous forms of LE.

In order to rigorously evaluate the methodological quality of the studies included in our systematic review, we employed the Newcastle-Ottawa Scale (NOS). This scale is designed to assess the quality of non-randomized studies, particularly focusing on three critical aspects: the selection of study groups, the comparability of these groups, and the ascertainment of the outcome of interest. Studies were awarded up to four stars for selection, two stars for comparability, and three stars for outcome. This comprehensive evaluation allowed us to quantitatively measure each study's quality, thereby ensuring the robustness of our review findings. Table 2 summarizes the NOS assessment for each study, providing a transparent overview of their methodological strengths and limitations.

**Table 2:** Quality assessment of selected studies using the Newcastle-Ottawa scale NOS: Newcastle-Ottawa Scale Selection: Assesses the method of selecting study groups and the ascertainment of exposure (for cohort studies). Comparability: Evaluates the comparability of cohorts or cases/controls based on design or analysis (e.g., controlling for confounding factors). Outcome: Examines how the outcomes are assessed, follow-up adequacy, and cohort follow-up adequacy.

First Author (Year)	Study Design	Selection (Max 4 Stars)	Comparability (Max 2 Stars)	Outcome (Max 3 Stars)	Total NOS Score (Max 9 Stars)	Key Quality Notes
Shangzhu Zhang [4] (2021)	Retrospective multicenter study	★★★★	★★	★★	8/9	Comprehensive data collection and analysis.
Sern Chin Lim [5] (2021)	Retrospective study	★★★★	★★	★★	8/9	Detailed clinical and imaging data, but retrospective.
Nuntana Kasitanon [8] (2002)	Retrospective study	★★★	★	★★	6/9	Good coverage of clinical characteristics but potential selection bias.
Filippa Nyberg [9] (2008)	Prospective cohort study	★★★★	★★	★★★	9/9	Strong methodology and detailed follow-up.
Hanan Al Rayes [10] (2002)	Retrospective study with prospective follow-up	★★★	★★	★★	7/9	Long-term follow-up, but small sample size.
B Tebbe [11] (1997)	Multicenter retrospective analysis	★★★	★★	★	6/9	Extensive sample, but retrospective limitations.

Discussion

This study has rigorously synthesized and analyzed the existing literature to provide a comprehensive overview of both dermatological and neurological manifestations in Systemic Lupus Erythematosus (SLE). The interplay between these two aspects, as evidenced in our findings, offers a deeper understanding of SLE's complex pathophysiology and its variable clinical presentation. This aligns with our aim to deepen the understanding of these intersecting domains and their implications for patient prognosis.

The integration of findings from various studies highlights the multifactorial nature of SLE, a key aspect of our objective to consolidate and critically evaluate the literature. For instance, the neuropsychiatric challenges outlined in studies by Zhang [4] and Lim [5] complement the dermatological insights provided by Nyberg [9] and Tebbe [11], illustrating the systemic nature of SLE. This holistic view is crucial for advancing our understanding of SLE and forms the basis for developing more effective, patient-centered treatment strategies.

Neuropsychiatric SLE, as illustrated in the studies by Zhang [4] and Lim [5], presents a myriad of challenges due to its diverse clinical presentations, ranging from seizures to acute confusional states. These manifestations underscore the need for early, accurate diagnosis and targeted therapeutic interventions. The study by Kasitanon et al. [8] further enriches this perspective by highlighting the efficacy of corticosteroids and anti-convulsants in managing specific neuropsychiatric symptoms, indicating the importance of individualized treatment strategies in SLE management.

In light of the crucial interconnection between dermatological and neurological manifestations in Systemic Lupus Erythematosus (SLE), it is important to recognize the potential overlap and influence these two aspects have on each other. The studies by Nyberg [9] and Tebbe [11], while primarily focusing on dermatological outcomes, implicitly suggest a complex interplay with neurological symptoms. For instance, skin manifestations in SLE, such as those detailed in these studies, can often be accompanied by or precede neuropsychiatric symptoms, hinting at a shared pathophysiological basis. This observation aligns with the broader understanding that SLE is a systemic condition where immune dysregulation can manifest in multiple organ systems simultaneously or sequentially. The potential for dermatological symptoms to serve as precursors or indicators of neurological involvement underscores the need for a vigilant, multidisciplinary approach to monitoring SLE patients. Recognizing these connections not only aids in early diagnosis but also in the formulation of comprehensive treatment plans that address the multifaceted nature of the disease. This integrated perspective is crucial for advancing our understanding of SLE and improving patient outcomes, as it allows for a more nuanced appreciation of how different disease manifestations can inform and influence each other.

Joerg Wenzel's research [12] further elucidates the pathogenesis of Cutaneous Lupus Erythematosus (CLE), emphasizing the role of interface dermatitis and its underlying immunological mechanisms. This understanding is pivotal in identifying novel therapeutic targets, such as specific cytokines and chemokines, and tailoring treatment strategies to mitigate skin involvement in SLE.

Our review resonates with the broader literature, including insights from Jarukitsopa et al. [7], Bertsias et al. [2], and Kivity et al. [3], highlighting the variable impact of SLE on patients' lives. The complex interplay of autoantibodies and cytokines, as discussed by Aringer et al. [13], underscores the need for a deeper understanding of the disease's pathogenesis to develop novel biomarkers and targeted therapies. This approach is crucial, especially in light of the correlation between high disease activity, specific antibody profiles, and the manifestation of NPSLE.

In addressing the long-term outcomes of SLE, our review particularly highlights concerns in cases with mixed connective tissue disease (MCTD). As discussed by Al Rayes et al. [10], the identification of complications such as pulmonary hypertension in these patients is crucial for both prognostication and management. This points to the need for careful monitoring and comprehensive care strategies, especially given the diverse and potentially severe implications of SLE and its associated conditions.

Building upon this, our review contributes significantly to the objective of enhancing patient care within the context of SLE. Through the examination of long-term prognosis, particularly focusing on the implications of dermatological and neurological symptoms, we underscore the importance of early detection and individualized treatment plans. The insights drawn from the broader literature and specific studies like those by Kasitanon et al. [8] and Wenzel [12] emphasize the heterogeneity of SLE manifestations. This variability necessitates a versatile and responsive approach to treatment, where the unique symptoms and progression of the disease in each patient are carefully considered.

Consequently, the findings of our review advocate strongly for an integrated, patient-centered approach to treating SLE, as highlighted by Fanouriakis et al. [6]. This approach is pivotal, considering the importance of simultaneously managing dermatological and neurological aspects to optimize patient outcomes. The necessity for interdisciplinary cooperation in treatment planning is apparent, advocating for a holistic care model that embraces the multifaceted nature of SLE. Such a model not only addresses the immediate clinical needs of patients but also takes into account the broader impact of the disease on their long-term health and quality of life.

This comprehensive review has illuminated the intricately connected and multifaceted nature of dermatological and neurological manifestations in Systemic Lupus Erythematosus (SLE) and their profound impact on patient prognosis. Through a meticulous synthesis of existing literature, we have enhanced the understanding of these complex interrelations, underscoring the necessity for an integrated, multidisciplinary approach in both research and clinical practice. The insights gained from this review, drawing on key studies [4,5,8,10,12,13], emphasize the need for innovative, patient-centered treatment strategies that consider the entire spectrum of SLE manifestations. As we look toward the future, it is imperative that research continues to delve deeper into the intricate relationship between the dermatological and neurological aspects of SLE. This pursuit should aim at uncovering novel therapeutic avenues, refining treatment protocols, and ultimately paving the way for more effective management strategies. By doing so, we not only address the unique challenges presented by SLE but also open the door to improving the quality of life for those affected by this complex and unpredictable disease.

## Conclusions

This systematic review has comprehensively explored the complex interplay between dermatological and neurological manifestations in SLE and their impact on long-term prognosis. Our findings underscore the multifaceted nature of SLE, highlighting the need for an interdisciplinary approach to diagnosis, treatment, and management. The studies reviewed reveal a significant correlation between disease activity and the severity of neuropsychiatric and cutaneous symptoms, emphasizing the importance of early detection and tailored treatment strategies. Future research should focus on developing integrated care protocols that address both dermatological and neurological aspects of SLE, aiming to improve overall patient outcomes and quality of life. Through this review, we aim to contribute to a deeper understanding of SLE, facilitating advancements in patient care and guiding future investigations in this field.
